# Editorial: Cell-Free Synthetic Biology

**DOI:** 10.3389/fbioe.2021.799122

**Published:** 2021-11-29

**Authors:** Jian Li, Yong-Chan Kwon, Yuan Lu, Simon J. Moore

**Affiliations:** ^1^ School of Physical Science and Technology, ShanghaiTech University, Shanghai, China; ^2^ Department of Biological and Agricultural Engineering, Louisiana State University, Baton Rouge, LA, United States; ^3^ Department of Chemical Engineering, Tsinghua University, Beijing, China; ^4^ School of Biosciences, University of Kent, Canterbury, United Kingdom

**Keywords:** cell-free systems, synthetic biology, cell-free protein synthesis, cell-free metabolic engineering, cell-free sensors, synthetic cells, biocatalysis and biotransformation

Cell-free systems have historically been used to decipher the genetic code and elucidate key principles of biological systems (Silverman et al., 2020; Garenne et al., 2021). Recently, renewed scientific interest in cell-free systems has driven the rapid development of cell-free synthetic biology, including the establishment of new cell-free platforms, bottom-up design of genetic circuits and artificial cells, prototyping of metabolic pathways, and designing of medical diagnostics and biosensors, among others (Lu, 2017; Moore et al., 2017; Liu et al., 2019; Copeland et al., 2021). Today, with the renaissance of cell-free biotechnology, more and more scientists are entering this field to explore the frontiers of synthetic biology. To highlight the current progress of cell-free research, this Research Topic aims to bring together diverse and exciting achievements in cell-free synthetic biology.

Here, we collected a total of 17 papers. These papers present a broad range of cell-free research across the development of new cell-free protein synthesis (CFPS) systems and construction of an educational “Genetic Code Kit.” The papers published in this topic are briefly introduced below.

Laohakunakorn et al. highlighted the current capabilities and challenges in the rapid growing field of cell-free synthetic biology. Due to the open nature of cell-free systems and breakthroughs in enabling technologies, bottom-up construction of increasingly complex biomolecular networks using cell-free systems will help address a wide range of fundamental biological questions such as the design of minimal life.

Kelwick et al. introduced key concepts and recent advancements in cell-free synthetic biology, with a focus on examples relevant to the materials sciences. In particular, the biological materials of industrial and societal importance manufactured by cell-free systems were summarized. While some challenges still remain, cell-free synthetic biology together with interdisciplinary collaborations and emerging technologies will lead to a new frontier for sustainable cell-free materials production and the growing bioeconomy.

Wu et al. summarized recent progresses on the efficient and extensive incorporation of non-canonical amino acids into proteins using CFPS. Different methods were used to increase the incorporation efficiency by elimination of endogenous competition factors, engineering of protein translation factors, and *in vitro* aminoacylation.

McSweeney and Styczynski reviewed the strategies of using linear DNA as expression templates in CFPS systems. Using linear DNA templates has some advantages, however, they can be quickly degraded by native exonucleases present in the cell extracts. This review described several approaches to stabilize linear DNA and compared their effectiveness and limitations, as well as future developments and applications.

Müller et al. introduced the modelling approaches and applications of CFPS systems, which can lay the foundation for understanding the production and degradation dynamics of macromolecules in CFPS. With the help of CFPS models, bottlenecks in the transcription and translation processes can be identified and key kinetic parameters can be determined from the modelled data, to aid the future development of enhanced CFPS systems.

Two papers discussed synthetic cells with different focuses. Damiano and Stano presented their opinion on the “life-likeness” of synthetic cells. Based on previous work, they promoted an “organizational approach” to the assessment of life-likeness and proposed the transition from behavioral assays (e.g., Turing test) to systemic strategies according to concepts like organization, complexity, networks, and emergence. Abil and Danelon pointed out in their perspective article that life is more than the sum of its constituted parts. These authors proposed a semi-rational, system’s level evolutionary approach for bottom-up building a synthetic cell.

Lin et al. developed a flow platform by combining CFPS systems and a tube-in-tube reactor that can measure and analyze the effect of oxygen on protein synthesis. Using this platform, energy, transcription, translation, and pathway networks in the reaction system were analyzed. The results showed that a maximum protein yield could be obtained with 21% oxygen, which is the same as the natural atmosphere oxygen condition.

Zhang et al. established a eukaryotic *Pichia pastoris*-based CFPS system. After a systematic optimization, this cell-free system was able to synthesize 50 μg/ml of protein, which is comparable to other eukaryotic CFPS platforms. As a result, it is a valuable addition to the current CFPS toolbox and will provide an alternative for synthesizing complex proteins requiring post-translational modifications.

Williams et al. reported the transformation of the research-based CFPS biotechnology into a hands-on module called the “Genetic Code Kit” for implementation into teaching laboratories. This kit can help improve students’ understanding of transcription and translation and prepare undergraduate students for careers in laboratory research.

Kim et al. reported the production of monomeric filaggrin, a human therapeutic protein, using an *E. coli*-based CFPS system. By relieving the limiting factors in transcription and translation, both protein yield and solubility were significantly improved with the enhanced CFPS system. This work demonstrates the potential of the *E. coli* CFPS to be adapted for studying therapeutic proteins.

Yang et al. designed modular cell-free systems for tunable biosynthesis of value-added chemicals. This approach was able to build long metabolic pathways (e.g., 6-7 enzymes) *in vitro* for defined biotransformation. Using two aromatic compounds (sharing the same upstream intermediate styrene) as examples, modular CFPS systems showed more than 80% conversion rates based on the same starting substrate of L-phenylalanine.

Arce et al. investigated cell-free RNA sensors using in-house made cell extracts instead of the commercial PURE CFPS system. CRISPRi-based technology was used to engineer the source strain to obtain nucleases silenced lysates, enabling CFPS from linear PCR-derived templates with high stability in the reaction. The home-made cell lysates were cost-effective and capable of virus detection using cell-free RNA toehold sensors.

Yue et al. described a systematic method for the expression and functional characterization of aquaporins based on an *E. coli* CFPS system, including template design, aquaporin expression and purification, proteoliposome preparation, and an aquaporin water permeability assay. This protocol can be adapted for the preparation of samples involving other channel proteins or transporters for their function assays.

Heide et al. provided a detailed protocol for the stepwise development and optimization of a CFPS platform derived from mammalian Chinese hamster ovary (CHO) cells. With the optimized CHO CFPS system, the protein yields were significantly improved, making it a useful research tool and a rapid production and screening platform for therapeutic protein development.

In addition to the above described cell lysates based cell-free systems, two research papers report the use of purified enzymes to implement *in vitro* biocatalysis. Fehlau et al. constructed a one-pot enzymatic cascade reaction for the synthesis of natural and modified nucleotides. Especially, a (d)ATP regeneration system was coupled in the reaction, leading to high-yielding production of nucleotides from nucleosides with high conversion rates. Liu et al. utilized T4 phage capsid as a protein scaffold for the immobilization of a three-enzyme cascade through SpyTag/SpyCatcher pairing. By doing this, the spatially organized enzymes exhibited higher enzymatic activity than the free enzymes, demonstrating the naturally occurring T4 phage scaffold is adaptable for multi-enzyme cascade assembly with enhanced biocatalytic activity.

In summary, the articles collated in this Research Topic demonstrate the potential of cell-free synthetic biology for a wide range of applications. Looking forward, cell-free synthetic biology research will continue to expand further to address current challenges in the field of synthetic biology when cell-based platforms are difficult or not amenable. With that, we now launch the second Research Topic on “Cell-Free Synthetic Biology, Volume II” and warmly welcome your significant contribution and submission.
